# Acute Gastric Dilatation Complicated by Necrosis and Perforation Following a Binge Eating Episode

**DOI:** 10.7759/cureus.31727

**Published:** 2022-11-21

**Authors:** Fabian Jano, Christopher Behr, Fawwaz Almajali, Vicki Moran

**Affiliations:** 1 Surgery, Saint Louis University School of Medicine, St. Louis, USA; 2 Trauma, Saint Louis University School of Medicine, St. Louis, USA; 3 Trauma, Saint Louis University Trudy Busch Valentine School of Nursing, St. Louis, USA

**Keywords:** laparotomy, binge eating, necrosis, perforation, gastric dilatation

## Abstract

Acute gastric dilatation is a rare entity limited to case reports in the literature. It is characterized by significant distention of the stomach beyond physiologic parameters. The sequelae of this phenomenon are life-threating, as it can lead to gastric perforation. It is frequently reported in patients who suffer from eating disorders, particularly binge eating disorder and bulimia nervosa. We present a case of a 28-year-old female who suffered from acute gastric dilatation following significant food intake. Her long hospital course was complicated by gastric necrosis and perforation, requiring multiple laparotomies prior to the restoration of a functional gastrointestinal tract. We aim to demonstrate the true gravity of this diagnosis and raise its awareness.

## Introduction

Acute gastric dilatation is a rare phenomenon characterized by abdominal distention, pain, and vomiting. These findings are best supported by radiological evidence, of which computed tomography (CT) is currently the best modality. Imaging typically demonstrates a severely distended stomach containing fluid or gas.

Several case reports have documented acute gastric dilatation in anorexic or bulimic patients, attributing the cause to episodes of significant binge eating [1]. However, due to a lack of accurate definition in the literature and overall lack of awareness, it is often missed early in its presentation [2]. Initial treatment consists of nasogastric decompression [3]. Signs of gastric necrosis or perforation warrant immediate surgical intervention due to the high risk of morbidity and mortality associated with a delay in treatment [1].

We present a patient who suffered from acute gastric dilatation that resulted in gastric necrosis and perforation.

## Case presentation

A 28-year-old woman with no reported past medical or surgical history was transferred to the emergency department from an urgent care clinic for diffuse abdominal pain. The pain started three days prior to presentation and progressively worsened. She had constant nausea, inability to vomit, and obstipation for two days. Prior to pain onset, she reported an episode of significant binge eating, having at least five meals within three hours at a food festival. The patient's discharge summary from the urgent care clinic included a CT scan impression reporting a severely distended stomach.

**Figure 1:**
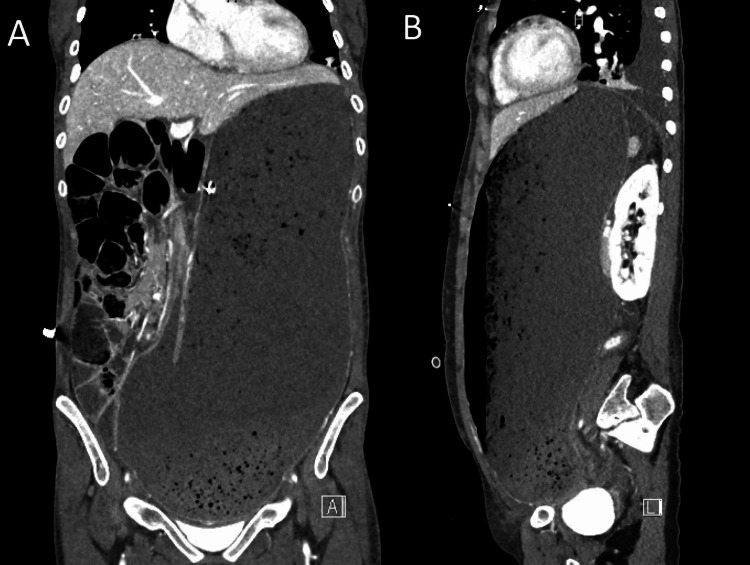
Coronal (A) and sagittal (B) abdominal CT scans showing acute gastric dilatation. The stomach is severely distended with an air-fluid level reaching the pelvic floor.

In the emergency department, her vital signs were within normal limits, and her physical exam was notable for a firm, distended, and diffusely tender abdomen without peritonitic signs. Laboratory analyses were notable for a leukocyte count of 13,700 cells/µL, sodium of 150 mmol/L, bicarbonate of 19 mmol/L, creatinine of 0.99 mg/dL, and lactate of 5.1 mmol/L. The patient received intravenous fluids and a nasogastric tube was inserted, which yielded approximately 7 L of gastric contents. Due to the inability to access the imaging performed at the urgent care, a CT scan was performed, which confirmed the previously reported massive gastric distension (Figure 1). She subsequently left against medical advice due to financial concerns.

**Figure 2:**
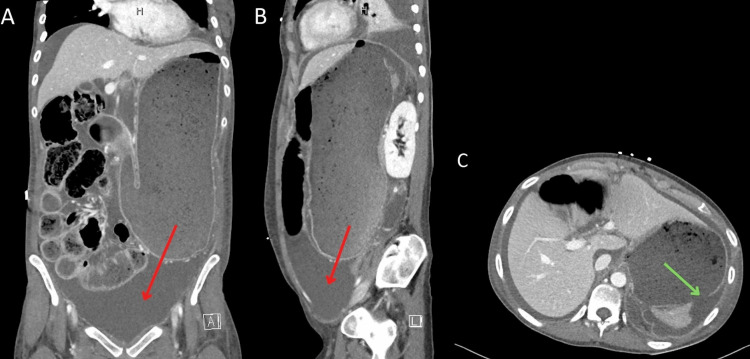
Coronal (A), sagittal (B), and axial (C) abdominal CT scans showing gastric perforation (green arrow) with intraperitoneal spillage of contents (red arrows).

The patient returned after six hours with increasing abdominal pain. She was ill-appearing and tachycardic at 160 bpm. Her exam was notable for a rigid abdomen with evident peritonitis. Given the significant change in her exam, a repeat CT scan was performed, which demonstrated gastric perforation with spillage of contents into the abdominal cavity (Figure 2). Lactic acid up trended to 12.26 mmol/L, raising concern for gastrointestinal ischemia. Pain management, fluid resuscitation, and broad-spectrum antibiotics were provided. She was subsequently taken for an exploratory laparotomy.

A midline laparotomy was performed, during which at least 5 L of food particles were removed from the abdominal cavity. The bowel appeared intact from the duodenum to the descending colon. A greater than 3 cm perforation was noted from the proximal greater curvature of the stomach to the spleen. The perforation margins were unclear due to the obliteration of the plane between the stomach and the spleen. Thus, a rescue splenectomy was performed to further expose the margins, followed by a partial gastrectomy using a linear cutting stapler. Additionally, there were three small areas of focal necrosis that were oversewn to prevent further perforation. Due to intraoperative shock, we elected to proceed with an open abdomen negative pressure therapy with plans to return the patient to the operating room once more stable. The patient remained intubated and was admitted into the surgical intensive care unit.

The patient required five further exploratory laparotomies due to the complications of the initial insult. On postoperative day one, the initial gastrectomy was revised due to the presence of necrosis around the staple line. Additionally, the previously oversewn focal areas of necrosis were excised and closed with sutures. The subsequent operations were directed at closing the abdomen but were complicated by extensive retroperitoneal edema and the inability to approximate the abdominal fascia. A Wittmann patch was placed on postoperative day seven to aid with the approximation of the abdominal fascia. On postoperative day 11, the eventual closure of the abdomen was achieved with the placement of gastrostomy and jejunostomy tubes.

The pathology report from the gastrectomy samples revealed acute mucosal hemorrhage with associated necrosis and ulceration. It was negative for any evidence of chronic gastritis, malignancy, or *Helicobacter pylori* infection.

The patient initially received nutrition via trophic feeds along with peripheral and total parenteral nutrition for a total of nine days. Parenteral nutrition was used due to the close proximity of the operations and the inability to maintain adequate nutrition via trophic feeds. She was eventually transitioned off parenteral nutrition to tube feeding on postoperative day 19 and eventually regular diet prior to discharge on postoperative day 28.

Psychiatry was consulted during the admission to evaluate for an eating disorder. Collateral history revealed a habit of heavy eating followed by prolonged periods of fasting. The patient was eventually diagnosed with an unspecified feeding and eating disorder and outpatient management was recommended.

## Discussion

The first documented case of acute gastric dilatation was by S.E. Duplay in 1833. Since then, there have been multiple case reports demonstrating this phenomenon [4]. Mechanical causes of gastric dilatation range from pyloric stenosis and volvulus to any process that physically obstructs gastric flow such as superior mesenteric artery syndrome. Non-mechanical causes include diabetic gastroparesis, bulimia and anorexia nervosa, postoperative gastric distension, electrolyte abnormalities, and medications [2]. Documented complications of the condition include perforation, gastric necrosis, compression of the aorta, and abdominal compartment syndrome [1,3,5,6]. We present an extreme case of acute gastric dilatation that was complicated by gastric necrosis, perforation, and shock. The sheer size of the dilatation, which occupied most of the abdominal cavity and where greater than 12 L of food particles were retrieved, demonstrates the severity of this case.

The mechanism of acute gastric dilatation is a subject of debate [3]. The incidence of acute gastric dilatation post-binge eating is well documented in case reports [1,5]. It is theorized that in bulimic patients, prolonged starvation leads to atrophy of the gastric musculature due to atony. When faced with massive food intake during a binge eating episode, the atrophic gastric musculature is unable to anterogradely push the gastric contents and is similarly unable to retrogradely push to induce vomiting [2,3,7]. Acute gastric dilatation has been implicated in non-large volume refeeding in the setting of malnutrition, which further supports the theory that gastric musculature atrophy is a likely cause of this phenomenon [7]. This mechanism is consistent with our patient’s scenario given a history of heavy eating followed by prolonged periods of starvation and the induction of severe dilatation after a binge eating episode. Interestingly, the lack of vomiting in our patient is unusual in so far as 90% of reported cases of acute gastric dilatation present with vomiting [1]. Our patient may not have been vomiting due to several possibilities, such as the previously discussed gastric muscular atrophy mechanism. Additionally, it is proposed that a “one-way valve” phenomenon can occur where food is only able to enter the stomach due to a distended gastric fundus blocking the gastroesophageal junction [7]. Bulimics have also been shown to have increased gastric capacitance, which can allow for significant distention beyond normal limits [8]. This would explain why the patient was able to retain a significant volume of food in her stomach. The superior mesenteric artery syndrome is another proposed mechanism contributing to acute gastric dilatation. Anorexic patients are predisposed to having diminished fat between the neurovascular bundle of the superior mesenteric artery and the third part of the duodenum, leading to duodenal compression and subsequent backup of gastrointestinal contents [7]. It is unclear whether our patient exhibited anorexia. While she qualified for an eating disorder, her body mass index (BMI) on admission was 20.53 kg/m2, including 11 L of undigested food. Her BMI on discharge was 15.65 kg/m2, which does qualify her for anorexia, but the patient had a prolonged hospital course and limited nutrition.

Ischemic necrosis of the stomach is unusual due to its extensive vasculature and collateral circulation [3]. However, several documented case reports of acute gastric dilatation have resulted in gastric necrosis [3-5]. It has been postulated that the intraluminal pressure must exceed venous pressure to induce gastric necrosis in humans [5]. Human cadavers have been shown to withstand up to 4 L of fluid prior to rupture [2,3,6]. In our patient, we hypothesize that the increased gastric intraluminal pressure compromised the vascular supply and led to ischemia with subsequent necrosis and perforation.

Acute gastric dilatation requires urgent diagnosis as early treatment can significantly reduce mortality [4]. If no signs of peritonitis are present, early nasogastric decompression helps reduce the intraluminal pressure and the risk of necrosis [1]. Even partial relief of volume could help prevent necrosis and induce vomiting if the gastroesophageal junction was initially obstructed [3]. Delayed necrosis is a possibility after decompression, warranting close observation [2]. Our patient did in fact have delayed perforation following nasogastric tube decompression. If perforation is ruled out, an upper endoscopy can aid with the etiology of the dilatation and can assess for mucosal ischemia. If extensive necrosis is suspected based on an upper endoscopy, urgent surgical intervention is warranted [3]. The treatment consists of resecting the necrotic tissue, resulting in a partial or complete gastrectomy. In cases of total gastrectomy, esophagojejunostomy and cervical esophagostomy may be required. A feeding jejunostomy should also be placed in consideration of the patient remaining *nil per os* in a long recovery period [1,4]. While our patient only required a partial gastrectomy, a rescue splenectomy was needed to hasten the procedure.

In the United States, the lifetime prevalence of binge eating disorder and bulimia nervosa are 1% and 2.6%, respectively [9]. Thus, these conditions are not uncommon, and given their association with acute gastric dilatation, further awareness is needed to help clinicians with the diagnosis of an acute abdomen in such a demographic.

## Conclusions

Acute gastric dilatation is a rare but life-threatening phenomenon that is limited to case reports in the literature. It is associated with eating disorders and carries a high mortality rate in cases that perforate. Conservative treatment in non-perforated cases involves nasogastric decompression. We present a case of acute gastric dilatation following a binge eating episode that resulted in gastric necrosis and perforation, requiring multiple operations and a prolonged hospital stay. We aim to spread awareness of this phenomenon to facilitate its timely diagnosis.
